# Concentrations of Polychlorinated Biphenyls and Organochlorine Pesticides in Umbilical Cord Blood Serum of Newborns in Kingston, Jamaica

**DOI:** 10.3390/ijerph13101032

**Published:** 2016-10-21

**Authors:** Mohammad H. Rahbar, Maureen Samms-Vaughan, Manouchehr Hessabi, Aisha S. Dickerson, MinJae Lee, Jan Bressler, Sara E. Tomechko, Emily K. Moreno, Katherine A. Loveland, Charlene Coore Desai, Sydonnie Shakespeare-Pellington, Jody-Ann Reece, Renee Morgan, Matthew J. Geiger, Michael E. O’Keefe, Megan L. Grove, Eric Boerwinkle

**Affiliations:** 1 Division of Epidemiology, Human Genetics, and Environmental Sciences (EHGES), University of Texas School of Public Health at Houston, Houston, TX 77030, USA; Jan.Bressler@uth.tmc.edu (J.B.); Eric.Boerwinkle@uth.tmc.edu (E.B.); 2 Division of Clinical and Translational Sciences, Department of Internal Medicine, University of Texas McGovern Medical School at Houston, Houston, TX 77030, USA; MinJae.Lee@uth.tmc.edu; 3 Biostatistics/Epidemiology/Research Design (BERD) Component, Center for Clinical and Translational Sciences (CCTS), University of Texas Health Science Center at Houston, Houston, TX 77030, USA; Manouchehr.Hessabi@uth.tmc.edu (M.H.); adickerson@hsph.harvard.edu (A.S.D.); 4 Department of Child & Adolescent Health, The University of the West Indies (UWI), Mona Campus, Kingston 7, Jamaica; msammsvaughan@gmail.com (M.S.-V.); cooredesai@googlemail.com (C.C.D.); sydonniesp@gmail.com (S.S.-P.); jodyreece@yahoo.com (J.-A.R.); renee.n.morgan@gmail.com (R.M.); 5 Human Genetics Center, University of Texas School of Public Health at Houston, Houston, TX 77030, USA; Megan.L.Grove@uth.tmc.edu; 6 Division of Chemistry and Toxicology, Michigan Department of Health and Human Services (MDHHS), Lansing, MI 48906, USA; sara.tomechko@gmail.com (S.E.T.); MorenoE@michigan.gov (E.K.M.); geigerm@michigan.gov (M.J.G.); okeefem@michigan.gov (M.E.O.); 7 Department of Psychiatry and Behavioral Sciences, University of Texas McGovern Medical School at Houston, Houston, TX 77054, USA; Katherine.A.Loveland@uth.tmc.edu

**Keywords:** polychlorinated biphenyls (PCBs), organochlorine (OC) pesticides, newborns, Kingston, Jamaica

## Abstract

To date much of the biomonitoring related to exposure to polychlorinated biphenyls (PCBs) and organochlorine (OC) pesticides is from middle to high income countries, including the U.S., Canada and Europe, but such data are lacking for the majority of low to middle income countries. Using data from 64 pregnant mothers who were enrolled in 2011, we aimed to assess the concentrations of the aforementioned toxins in umbilical cord blood serum of 67 Jamaican newborns. For 97 of the 100 PCB congeners and 16 of the 17 OC pesticides, all (100%) concentrations were below their respective limits of detection (LOD). Mean (standard deviation (SD)) lipid-adjusted concentrations in cord blood serum for congeners PCB-153, PCB-180, PCB-206 and total PCB were 14.25 (3.21), 7.16 (1.71), 7.30 (1.74) and 28.15 (6.03) ng/g-lipid, respectively. The means (SD) for the 4,4′-dichlorodiphenyldichloroethylene (DDE)-hexane fraction and total-DDE were 61.61 (70.78) and 61.60 (70.76) ng/g-lipid, respectively. Compared to the U.S. and Canada, the concentrations of these toxins were lower in cord-blood serum of Jamaican newborns. We discuss that these differences could be partly due to differences in dietary patterns in these countries. Despite limitations in our dataset, our results provide information on the investigated toxins in cord blood serum that could serve as a reference for Jamaican newborns.

## 1. Introduction

Several organochlorine (OC) compounds, including polychlorinated biphenyls (PCBs) and selected chlorinated pesticides, are persistent organic pollutants of increased public health concern due to their bioaccumulative and toxic properties. Unlike the chlorinated phenols and the chlorophenoxyacetic acid compounds, the long half-lives and high sorption coefficients of PCBs and selected OC pesticides, such as dichlorodiphenyltrichloroethane (DDT) (its metabolite dichlorodiphenyldichloroethylene (DDE)) and chlordane, enable these chemicals to remain in the environment for several decades post-application and discontinuation. These compounds persist in numerous environmental media, including soil and water, and can eventually bioaccumulate and magnify in biological organisms, principally seafood [1], meat, poultry, eggs and dairy products [2,3], and ultimately in humans. Adverse health effects of PCBs and OC pesticides exposure have been reported and include male and female reproductive effects, endocrine disruption [4,5], carcinogenicity [4], neurotoxicity [4] and behavioral/developmental outcomes [4].

To date, much of the biomonitoring data related to exposure to PCBs and OC pesticides are from middle to high income countries, including the U.S., Canada and some European countries. For example, based on three surveys in the years 1973–1974, 1979–1982 and 1989–1991 that were conducted in the Great Lakes region of the U.S., Karmous et al. (2004) reported that more than 70% of mothers had a p,p′-DDE maternal serum concentration >5 µg/L (>5 ng/mL) and more than 95% had maternal serum total PCB concentrations >5 µg/L [6]. From a study in Brownsville, Texas, which involved 35 pregnant Hispanic women between October 2005 and February 2006, Sexton et al. (2013) reported that maternal and cord blood serum concentrations for 22 PCBs were ≤0.2 ng/mL, and concentrations of total PCBs (sum of all PCB congeners assessed) averaged more than 2.5 ng/mL [7]. Based on information from the Canadian Health Measures Survey (CHMS), it has been reported that geometric mean of the concentrations for total DDE was 0.91 ng/mL in maternal blood of Canadian females (20–39 years) who were studied during Cycle 1 (2007–2009) [8]. Moreover, in another study from Canada, Dallaire et al. reported that the arithmetic mean of total the PCB concentrations for 14 PCB congeners in cord blood plasma was 343.8 (geometric mean = 273.8 μg/kg) [9]. Govarts et al. reported that median cord blood serum PCB-153 and p,p′-DDE concentrations in the 15 populations studied in a multi-country European project ranged from 20–484 ng/L (0.02–0.484 ng/mL) and from 50–1208 ng/L (0.05–1.208 ng/mL), respectively [10]. A more recent study, from Rio Grande do Sul state, Brazil, which examined the levels of five PCB congeners (28, 52, 138, 153 and 180) in umbilical cord serum samples from 148 newborns reported that the sum of these PCBs ranged from 0.35–55.17 ng/mL and that PCB 138 was the most prevalent congener [11].

As an island nation, Jamaica has very specific sources of exposure to environmental contaminants, including PCBs and OC pesticides [12,13,14,15]. According to Fernandez et al. (2007), PCBs and OC pesticides have been used extensively in the Wider Caribbean Region (WCR), including Jamaica, since the 1930s [14]. Studies have been conducted to ascertain the levels of PCBs in soil, water, and sediments in some urban and rural areas in Jamaica and reported mean PCB concentrations in urban and rural soils of 1.4 mg/kg and less than 0.1 mg/kg, respectively [16]. The International Mussel Watch (IMW) project has assessed levels of PCBs and OC pesticides in bivalves in the North, Central and South American coasts [17], as well as in the WCR [18] and found that the most frequently-reported organic pollutants in the WCR include PCBs and OC pesticides [18]. While in a more recent report, the IMW indicated a decline in PCBs and OC pesticides in the coastal regions of the U.S. [17], a similar decrease has not been reported in the WCR. Notably, a 2012 report based on maternal blood samples from pregnant women from 10 Caribbean Communities (CARICOM) that participated in the Caribbean Eco Health Programme (CEHP) indicated lower levels of PCBs and OC pesticides compared with those in the U.S. and Canada [19].

The Pesticides Control Authority (PCA) of the Ministry of Health, which supervises the importation and use of pesticides in Jamaica [20], initiated a program in 2010 to collect unwanted and unused pesticides that people keep in their homes [21]. In 2010, an interview with the PCA Registrar in a local newspaper indicated that mitigating illegal importing of unregistered pesticides continues to be a major problem for Jamaican customs authorities [21]. In 2011, the PCA banned six other pesticides due to their widespread misuse and their potential harmful effects on health. The OC pesticides among these included chlordecone (kepone), which was never registered for use in Jamaica; endosulfan (thiodan), which has been used in the cultivation of coffee; and lindane (γ-HCCH (hexachlorocyclohexane) = γ-BHC), which was only registered for use on animals in Jamaica [22]. These reports highlight the need for the assessment of the exposure to PCBs and OC pesticides in the Jamaican population, particularly in newborns. In this study, we characterize the concentrations of select PCBs and OC pesticides in umbilical cord blood serum of newborns in Kingston, Jamaica.

## 2. Materials and Methods

### 2.1. Study Population

Data for this study were generated in collaboration with the faculty at the University of the West Indies (UWI), Mona Campus, in Kingston, Jamaica. The Jamaican Birth Cohort (JA Kids) Study is the second national birth cohort study of Jamaican children and is especially focused on development and behavior. This study enrolled about 9500 pregnant women during their prenatal period (third trimester), interviewed 5500 of them at delivery in 2011, and followed about 2000 children of these women to at least 2 years of age [23]. For a subsample of 144 mothers who delivered in the Kingston area, cord blood serum was isolated.

Participants in the study reported here comprise a subsample of 64 mothers with 67 newborns, including three sets of twins, for whom we had complete data. Demographic characteristics of the study population are displayed in Table 1. We used data from two questionnaires administered to mothers in their third trimester and at the time of delivery by the JA Kids study. Through these two questionnaires, we collected information regarding demographic and socioeconomic variables, including level of maternal education and assets owned by the family. Details regarding the information in these questionnaires was published previously [24]. Study data were collected and managed using Research Electronic Data Capture (REDCap) [25] hosted at the University of Texas Health Science Center at Houston (UTHealth). All serum analyses were performed by the Analytical Chemistry Section of the Michigan Department of Human Health Services (MDHHS), which has Clinical Laboratory Improvement Amendments (CLIA) and College of American Pathologist (CAP) accreditation. The study was conducted in accordance with the Declaration of Helsinki, and the protocol was approved by the Institutional Review Boards (IRBs) of UTHealth, UWI and MDHHS (Project Identification Code HSC-SPH-09-0059). All mothers gave their informed consent in compliance with the IRBs for inclusion before they participated in the study.

### 2.2. Cord Blood and Serum Collection

Cord blood samples (8–10 mL) were collected at delivery in 10-mL red top vacutainer tubes. Cord blood aliquots were allowed to clot for 30–60 min at ambient/room temperature, during which time they were transferred from the hospital to the Molecular Biology Lab of UWI with extra precautions to have as little agitation as possible. The resulting serum (~4 mL) was transferred into pre-labeled, hexane-rinsed glass vials with Teflon-lined screw caps that were provided by the Analytical Chemistry Section of MDHHS. Serum vials were frozen upright (at −10 °C down to −20 °C) within 90 min of the cord blood draw. Frozen serum samples were stored at −20 °C in the Molecular Biology Lab of UWI and later transported to MDHHS in Lansing, Michigan, using Cryoport^®^ dry vapor shippers (Irvine, CA, USA). 

### 2.3. Target Compounds, Reagents and Assessment of PCBs and OC Pesticides in Cord Blood Serum

Cord blood serum samples were analyzed for 100 of the 209 PCB congeners (listed in Table 2), including 13 dioxin-like PCBs (77, 105, 114, 118, 123, 126, 156, 157, 167, 169, 170, 180 and 189). Total PCBs is the sum of all 100 congeners, including three paired congeners (066/095, 138/163 and 196/203). In addition, cord blood serum samples were analyzed for the following 17 OC pesticides: DDT and its metabolites DDE and dichlorodiphenyldichloroethane (DDD) (4,4′-DDT (p,p′-DDT), 2,4′-DDT, 4,4′-DDE (p,p′-DDE), 4,4′-DDD and 2,4′-DDD), selected chlordane isomers and metabolites (α-chlordane, γ-chlordane and oxychlordane), select chlordane-related compounds and metabolites (trans-nonachlor, cis-nonachlor, heptachlor and heptachlor epoxide), select hexachlorocyclohexane (HCCH) isomers (β-HCCH (β-BHC) and γ-HCCH (γ-BHC/lindane)), hexachlorobenzene (HCB) and mirex. The analytes of interest are found in two distinct analytical fractions: the hexane fraction and the benzene fraction; total DDE is the sum of 4,4′-DDE from both. A detailed description of the laboratory analysis and quality control is provided as Supplementary Materials. The limits of detection (LODs) were established for each analyte using Equation (S1). The LOD for total PCB is the algebraic sum of the LODs of the three most common PCB congeners found in biomonitoring samples (153, 170 and 180). For total 4,4′-DDE, we used the LOD from the lowest fraction (i.e., hexane).

### 2.4. Statistical Analysis

We examined the distributions of various characteristics of the study sample, including demographic and socioeconomic status (SES). Using Equation (S2), we computed lipid-adjusted concentrations for PCBs and OC pesticides. If cord blood serum PCB and OC pesticide concentrations (or analyte concentrations) were below LOD, we replaced these by half of their respective LODs for computing lipid-adjusted concentrations [26]. We calculated the percentage of the values below LOD for all PCB and OC pesticide concentrations. We assessed means and standard deviations (SD) for lipid-adjusted PCB and OC pesticide concentrations. All statistical analyses were conducted using SAS 9.4 (Cary, NC, USA) [27].

## 3. Results

The mean age of mothers at delivery was 29.1 years with 55% below the age of 30 years. Nearly 58% of the newborns were female. About 68% of mothers reported previous pregnancies. Information regarding other demographic and socioeconomic characteristics of the study population is displayed in Table 1.

Notably, the PCBs and OC pesticide concentrations in almost all of the cord blood samples were below the LOD. For 97 of the 100 PCB congeners and 16 of the 17 OC pesticides that we assessed in this study, all (100%) of the concentrations were below their respective LOD. Mean concentrations for total PCB and total DDE were 0.0625 and 0.1344 ng/mL, respectively, with lipid-adjusted concentrations for total PCB and total DDE of 28.15 (6.03) and 61.60 (70.76) ng/g-lipid, respectively. The mean (SD) lipid-adjusted concentrations in cord blood serum for PCB congeners (153, 180, 206) and 4-4′-DDE-hexane fraction (p,p′-DDE) in this study were 14.25 (3.21), 7.16 (1.71), 7.30 (1.74) and 61.61 (70.78) ng/g-lipid, respectively. Additional information regarding the distributions of the lipid-adjusted PCB and OC pesticide concentrations are displayed in Table 2.

## 4. Discussion

In this article, we reported the lipid-adjusted PCB and OC pesticide concentrations in cord blood serum of Jamaican newborns, which we believe have not been previously published. Over 97% of the 100 PCB congeners and 94% of the 17 OC pesticides assessed had cord blood serum concentrations that were all (100%) below their respective LOD. For three PCB congeners (153, 180, 206), we had at least one concentration above the LOD. For only one OC pesticide, 4,4′-DDE, 37% of the concentrations were above the LOD; the mean concentration was 0.1344 ng/mL, which resulted in a mean (SD) lipid adjusted concentration of 61.60 (70.76) ng/g-lipid. 

### 4.1. PCBs and OC Pesticides Concentrations in Cord Blood Serum of Jamaican Newborns

CEHP was the first study that reported concentrations of PCBs and OC pesticides in maternal blood from CARICOM countries, including Jamaica. They reported geometric means for PCB and OC pesticide concentrations in maternal blood for six PCB congeners (118, 138,153, 156, 170, 180) and five OC pesticides (HCB, total DDE), heptachlor epoxide, trans-nonachlor and 4,4′-DDT, for which concentrations were detected in more than 40% of their samples. Although CEHP replaced half of the LOD for concentrations that were below LOD [19], we recognize that this method may not be the most appropriate for reporting measures with a large percentage of measurement below the LOD. Although a greater percentage of the samples in our study had PCB and OC pesticide concentrations below the LOD than that reported by CEHP, the geometric mean concentration of total DDE was much higher in CEHP compared with our study (0.63 vs. 0.10 ng/mL). Nevertheless, both the findings by CEHP related to concentrations of the aforementioned PCBs and OC pesticides in Jamaican maternal blood and our results from cord blood serum in Jamaican newborns reported here are lower than those of the U.S. and Canada [19]. For example, the geometric mean concentrations for total DDE from our study (i.e., 0.10 ng/mL) and CEHP (0.63 ng/mL) are both lower than the geometric mean concentrations for total DDE of 0.91 ng/mL and 1.69 ng/mL found in maternal blood of Canadian females (20–39 years) who were studied during CHMS-Cycle 1 (2007–2009) [8] and females (16–49 years) who participated in the NHANES 2001–2002 study in the U.S., respectively [19]. However, we acknowledge that the differences we have reported here could be partly due to the differences in the biological specimens used for these assessments (i.e., cord blood serum vs. maternal blood).

Another study from Brownsville, Texas, that collected both maternal and umbilical cord blood samples from 35 pregnant Hispanic women between October 2005 and February 2006 reported that maternal and cord blood serum concentrations for 22 PCBs were ≤0.2 ng/mL, and concentrations of total PCBs (sum of all PCB congeners assessed) averaged more than 2.5 ng/mL [7,28]. In addition, they reported that 11 OC pesticides/metabolites and nine PCBs concentrations were not detected in at least 75% of samples [28]. Overall, the results reported from the Brownsville study were higher than our results in Jamaica. 

A study from southern Italy that involved 32 matched pairs of pregnant women and cord blood serum of their 32 newborns reported that mean umbilical serum concentrations for 10 “dioxin-like” PCB congeners (77, 81, 105, 118, 123, 126, 156, 167, 169 and 189) were 0.23, 0.23, 0.05, 0.04, 0.54, 6.49, 0.32, 1.19, 1.26 and 0.77 ng/mL, respectively. However, our results from Jamaica for the same “dioxin-like” PCB congeners were 100% below the LODs of 0.25, 0.06, 0.06, 0.06, 0.06, 0.25, 0.03, 0.06, 0.6 and 0.03 ng/mL, respectively, indicating that concentrations of these PCBs are much lower in Jamaican newborns than those reported by the study from southern Italy [29]. Based on 148 Brazilian newborns, Mohr et al. (2015) reported mean umbilical cord serum concentrations of 3.83 and 1.55 ng/mL for PCB congeners 138 and 153, respectively [11]. These levels were much higher than those we observed in Jamaican newborns. Overall, the concentrations of PCBs and OC pesticides that we have reported from the Jamaican newborns are comparable to or lower than those published from other countries. 

Due to past use of OC pesticides in agriculture throughout the 1990s, particularly in coffee production, p,p′-DDE has been detected in soil, as well as animal and plant life in coastal waters, with 6.14 ng/g of p,p′-DDE measured in sediments from Portland between October 1991 and December 1992 [13] and 8.3 ng/g in shrimp in Hunts Bay and Kingston Harbor in 1995/1996 [12]. Although most OC compounds were banned for use in Jamaica in 1999 [13], the persistence of these toxicants allows for potential exposure in our unique study population. Compared to Jamaica [30], PCBs and some OC compounds were banned for use much earlier in the U.S. (1979) [31] and in Canada and Europe (1985) [32,33]. Therefore, compared to Jamaica, one may expect that levels of exposure to these toxins would be less in the U.S. and Canada. However, the lower concentrations seen in Jamaica as compared to the U.S. and Canada may indicate that regulatory measures in Jamaica have been effective in limiting these exposures.

### 4.2. Role of Dietary Sources of Exposure to PCBs and OC Pesticides as a Possible Explanation for Lower Concentrations in the Jamaican Population Compared with Other Countries

A major source of PCBs and OC pesticides is food including contaminated fish, meat, poultry, eggs and dairy products [1,2]. Dietary patterns are considered as important sources of exposure to PCBs and OC pesticides [4]. Although PCBs and OC pesticides are found in both meat and fish, the levels of PCBs and OC pesticides in meat and fish depends on the dietary patterns of animals and fish [2,34,35]. For example, in the U.K., food consumption accounts for 97% of the total PCB exposure (0.53 μg/person/day) [4]. Some foods, such as beef, contain lesser amount of PCBs than seafood, but in populations in which beef is consumed more than seafood annually, beef accounts for a higher percentage of PCBs in the total diet [36]. Exposure to these toxins is considered to be lower in plants (e.g., fruits and vegetables), as well as meat and dairy products from grazing animals compared to fish [2]. In addition, higher levels of PCBs are found in bottom-feeders, such as carp, than other fish [2,4]. A report from an Environmental Working Group indicated that farmed salmon is the most PCB-contaminated protein source in the U.S. food supply and has four-times the levels in beef and 3.4-times the dioxin-like PCBs found in other seafood [37]. In Jamaica, only 28% of total seafood consumed is domestic seafood [38]. On the other hand, salmon, crabmeat, scallops and squid are the main species imported from the U.S., but the majority of Jamaica’s seafood imports are low priced species from Canada and Norway that include mackerel, cod and sardines [38]. Although, compared to populations in the U.S. and Canada, the Jamaican population has a higher consumption of seafood, the types of seafood consumed have lower levels of PCBs. 

On the other hand, per capita consumption, as well as the type of fish and meat consumed by a population could allow comparison of the levels of exposure to PCBs and OC pesticides in populations with different dietary patterns. For example, in the U.S., the exposure reduction to PCBs due to a 12% reduction in beef consumption would be equal to the total amount of such exposure from 100% per capita consumption of salmon [36]. In the following, we will use available information from published reports from different countries to make comparisons with the Jamaican population. It has been reported that fish consumption in Jamaica is about twice that consumed in the U.S. and Canada. For example; fish consumption in Arctic Quebec is 3.4 fish meals/week [39] and for Jamaica is 5.5 fish meals/week [40]. In 2007, per capita fish consumption in Jamaica was 14.73 kg/person/year [41], but in 2010, for the U.S. population, per capita seafood consumption was about half that of Jamaica (7.2 kg/person/year or 15.8 pounds/person/year) in edible weight equivalent terms [42]. Moreover, for the U.S. in 2013, per capita consumption of chicken was nearly 57.7 pounds/person/year and for dairy products was over 600 pounds/person/year [43]. In contrast, in 2009, per capita meat consumption for Jamaicans was 59.1 kg/person/year, about half of the amount consumed in the U.S. (120.2 kg/person/year) and Canada (94.3 kg/person/year) [44]. Furthermore, Jamaicans consume lower amounts of milk and dairy products compared to the U.S. and Canada. For example, it has been reported that overall per capita consumption of milk remains at 105 mL/day, which is one-third of that for Latin America and the Caribbean; one-fifth of the average for developed countries; and half of the minimum amount recommended by the World Health Organization [45]. Since higher levels of exposure to PCBs and OC pesticides are associated with meat consumption than fish consumption [36], the differences in the consumption of the aforementioned food items could partly explain higher levels of PCBs and OC pesticide concentrations reported from cord-blood isolated for newborns in the U.S. and Canada, compared to those of Jamaican newborns.

## 5. Limitations

We acknowledge several limitations in this study. First, since information from the third trimester and delivery questionnaires was not available for some of the 64 mothers in this study, we have different levels of missing information for some of the variables reported here. We also acknowledge that all of the participants in our study delivered their babies in hospitals in the Kingston area. Therefore, the findings reported in this article may not be representative of all newborns in Jamaica. In this study, all PCB and OC pesticide concentrations that were below their respective LOD were replaced with half of the LOD when calculating lipid-adjusted measures. We acknowledge that the lipid adjusted values will be affected by the underlying assumptions made for the adjustment, which may result in chance variation. Therefore, the standard deviations for lipid adjusted concentrations reported here should be interpreted with caution. Other studies have suggested different estimation and imputation techniques depending on the percentage of measurements below LOD. For example, Lubin et al. (2004) advocated using imputations based on a Tobit regression model, which assumes a log-normal distribution when the percentage of measurements below the LOD is less than 80% [46]. However, we found that the Tobit regression model was not suitable for our situation where the percentage below LOD was nearly 100%. Our literature review indicated that the methodology is lacking for the imputation of values in our situation. We suggest the development of new methods for imputations that are based on the population distributions of the measurements in the geographic area of the study. Another limitation is that we discussed dietary intake in the aggregate (e.g., per capita consumption) because individual level data regarding dietary intake of the mothers were not available. Furthermore, due to the limited sample size, particularly the limited number of measurements above LOD, we did not conduct any inferential statistics in the present study.

## 6. Conclusions

In this study, we are the first to report lipid-adjusted concentrations of 100 PCB congeners and 17 OC pesticides in cord blood serum of Jamaican newborns, which could serve as a reference for Jamaican newborns. Our results indicate that cord blood serum concentrations of PCBs and OC pesticides in Jamaican newborns are similar to or lower than the levels reported for maternal blood in an earlier study from Jamaica. In addition, the cord blood serum concentrations of PCBs and OC pesticides in Jamaican newborns are similar to or lower than the levels reported for cord blood serum or maternal blood from other studies in the U.S. or Canada.

## Figures and Tables

**Table 1:** Individual and household characteristics of the study participants (*N* = 67 newborns from 64 mothers) from the Jamaican Birth Cohort (JA Kids) study.

Variable	Categories	*N* (%)
Sex of newborn *	Male	28 (41.8)
	Female	39 (58.2)
Maternal age (years) (at newborn’s birth)	Age < 25	13 (20.3)
	25 ≤ Age < 30	22 (34.4)
	Age ≥ 30	29 (45.3)
Maternal education (at newborn’s birth)	Up to high school	25 (39.1)
	Beyond high school	39 (60.9)
Previous pregnancies ^a^	Yes	41 (68.3)
	No	19 (31.7)
Assets owned by the family	TV	64 (100)
	Refrigerator	63 (98.4)
	Freezer ^b^	20 (40.8)
	Living room set ^c^	53 (88.3)
	Washing machine ^d^	49 (80.3)
	Cars or other vehicle	33 (51.6)
	Cable/satellite connection ^e^	49 (77.8)

* Since 3 pregnancies resulted in twins, data are presented for 64 mothers and 67 newborns; ^a^ previous pregnancies status was missing for 4 mothers; ^b^ ownership of freezer was missing for 15 families; ^c^ ownership of living room set was missing for 4 families; ^d^ ownership of washing machine was missing for 3 families; ^e^ Cable/Satellite connection was missing for 1 family.

**Table 2:** Lipid adjusted mean concentrations of PCBs and OC pesticides in cord blood serum samples of 67 Jamaican newborns from the JA Kids study.

Measure	N	% Below LOD	LOD (ng/mL)	Lipid-Adjusted Mean (SD) (ng/g-lipid)
**PCB Congener Number**				
128,137,156,157,158,170,172,175, 183, 185, 187, 190, 193, 194, 195, 198, 199, 200, 205	67	100	0.0313	7.05 (1.51)
179,189	66	100	0.0313	7.06 (1.52)
207	60	100	0.0313	7.1 (1.49)
22, 40, 48, 56, 60, 81, 82, 83, 87, 90, 97, 110, 118, 123, 130, 132, 134, 135, 141, 144, 151, 160, 167, 169, 171, 174, 177, 178, 182, 196/203, 201	67	100	0.0625	14.07 (3.01)
64,105, 114, 146	66	100	0.0625	14.09 (3.03)
63	57	100	0.0625	14.01 (3.07)
16, 18, 25, 26, 27, 32, 33, 37, 42, 44, 45, 47, 49, 52, 70, 71, 74, 84, 91, 92, 99, 100, 138/163, 149	67	100	0.125	28.15 (6.03)
17, 31, 101, 136	66	100	0.125	28.18 (6.07)
28	36	100	0.125	27.65 (5.8)
66/95, 77, 126	67	100	0.25	56.29 (12.05)
8	67	100	0.3125	70.36 (15.07)
11	67	100	0.625	140.75 (30.15)
1	61	100	0.625	141.69 (30.72)
3	9	100	0.625	153.62 (38.16)
153	66	98.5	0.0625	14.25 (3.21)
180	67	98.5	0.0313	7.16 (1.71)
206	57	98.2	0.0313	7.30 (1.74)
Total PCB ^a^	67	100	0.1250	28.15 (6.03)
**OC Pesticides (*Hexane Fraction*)**				
HCB ^b^, heptachlor, mirex	67	100	0.0625	14.07 (3.01)
4,4′-DDE ^c^	67	62.7	0.1250	61.61 (70.78)
**OC Pesticides (*Benzene Fraction*)**				
γ-chlordane, α-chlordane, oxychlordane, heptachlor epoxide, trans-nonachlor, cis-nonachlor, 2,4′-DDD ^d^, 2,4′-DDT ^e^ and 4,4′-DDT ^e^	67	100	0.1250	28.15 (6.03)
γ-HCCH ^f^, β-HCCH ^g^, 4,4′-DDE ^c^ and 4,4′-DDD ^d^	67	100	0.2500	56.29 (12.05)
Total-DDE ^h^	67	62.7	0.1250	61.60 (70.76)

^a^ LOD for total PCB is the algebraic sum of the LODs of the three most common PCB congeners found in biomonitoring samples (PCB153, PCB170 and PCB180); ^b^ HCB = hexachlorobenzene; ^c^ DDE = dichlorodiphenyldichloroethylene; ^d^ DDD = dichlorodiphenyldichloroethane; ^e^ DDT = dichlorodiphenyltrichloroethane; ^f^ γ-HCCH = gamma-hexachlorocyclohexane (γ-BHC/lindane); ^g^ β-HCCH = beta-hexachlorocyclohexane (β-BHC); ^h^ LOD for total 4,4′-DDE is the LOD from the lowest fraction.

## Supplementary Materials

The following are available online at www.mdpi.com/1660-4601/13/10/1032/s1.
