# Supplementary material for: Concentrations of Polychlorinated Biphenyls and Organochlorine Pesticides in Umbilical Cord Blood Serum of Newborns in Kingston, Jamaica
Source: Int J Environ Res Public Health. 2016 Oct 21;13(10):1032. doi: 10.3390/ijerph13101032 (PMC5086771; doi:10.3390/ijerph13101032)
Supplement: Supplementary file 1 [file ijerph-13-01032-s001.pdf]

# Supplementary Materials: Concentrations of Polychlorinated Biphenyls and Organochlorine Pesticides in Umbilical Cord Blood Serum of Newborns in Kingston, Jamaica

**Mohammad H. Rahbar, Maureen Samms-Vaughan, Manouchehr Hessabi, Aisha S. Dickerson, MinJae Lee, Jan Bressler, Sara E. Tomechko, Emily K. Moreno, Katherine A. Loveland, Charlene Coore Desai, Sydonnie Shakespeare-Pellington, Jody-Ann Reece, Renee Morgan, Matthew J. Geiger, Michael E. O'Keefe, Megan L. Grove and Eric Boerwinkle**

## 1. Target Compounds, Reagents and Assessment of PCBs and OC Pesticides in Cord Blood Serum

### *1.1. Reagents and Laboratory Analysis*

All solvents (methanol, diethyl ether, *n*-hexane, water, benzene and iso-octane) were of pesticide grade (J.T. Baker, Center Valley, PA, USA; Burdick & Jackson, Muskegon, MI, USA; Alfa Aesar, Ward Hill, MA, USA). Anhydrous sodium sulfate was solvent-rinsed and heated at 130 °C for at least 24 h prior to use (J.T. Baker, Center Valley, PA, USA); Florisil® was also heated at 130 °C for at least 24 h prior to use (EMD, Gibbstown, NJ, USA). A three percent (3%) deactivated silica gel was prepared in-house and was used within five days of preparation (EMD, Gibbstown, NJ, USA). Calibration and verification standards were prepared in-house from custom analytic standards of PCB congeners and OC pesticides (AccuStandard, New Haven, CT, USA; Ultra Scientific, N. Kingstown, RI, USA). The surrogate recovery spiking solution was obtained as a custom analytic standard (Ultra Scientific, N. Kingstown, RI, USA).

The analytes of interest were extracted from the serum samples using a liquid-liquid extraction approach based on modifications to Environmental Protection Agency (EPA) and CDC methodology [1–6]. Serum samples (~4 mL) were spiked prior to extraction with surrogate solution for recovery assessment. Methanol was added to the surrogate-spiked serum for protein precipitation, followed by extraction with 1:1 *n*-hexane:diethyl ether. The extracts were condensed to dryness for gravimetric lipid determination and were then re-suspended in *n*-hexane prior to cleanup and fractionation. Extract cleanup and matrix removal was achieved using Florisil® column chromatography. The Florisil® eluents were then separated into two analytical fractions using 3% deactivated silica gel. The two resulting fractions from the serum extracts (hexane and benzene) contained the PCB congeners and OC pesticide analytes of interest and were analyzed by gas chromatography with electron capture detection (GC-ECD).

The hexane fractions contained the PCB congeners and select OC pesticides and were analyzed on an Agilent 7890A (7693 autosampler) and a Varian CP-3800 (8400 autosampler), both of which were equipped for dual capillary column analyses with 63Ni detectors and the following columns: DB-5 (60 m × 0.2500 mm × 0.2500 µm) and DB-1701 (60 m × 0.2500 mm × 0.2500 µm) (J & W Scientific, Santa Clara, CA, USA). The instrument conditions were as follows: injector(s) at 280 °C, carrier gas (hydrogen) at 33 psi (constant pressure), detectors at 320 °C and make-up gas flow (nitrogen) at 25 mL/min. The oven temperature program was as follows: injection at 110 °C (hold for 1.5 min), ramp temperature by 10 °C/min to 170 °C (no hold), ramp temperature by 1.8 °C/min to 230 °C (no hold) and, finally, ramp by 3 °C/min to 300 °C (hold for one minute). The analytical signals were recorded by the following chromatography software: OpenLAB CDS ChemStation Edition (Agilent Technologies) [7] and Galaxie™ (CompassCDS) [8].

The benzene fractions contained the remaining OC pesticides and were analyzed on an Agilent 7890B (7693 dual injector), equipped for dual capillary column analyses with 63Ni detectors and the following columns: DB-XLB (30 m × 0.2500 mm × 0.50 µm) and DB-35MS (30 m × 0.2500 mm × 0.2500 µm) (J&W Scientific, Santa Clara, CA, USA). The instrument conditions were as follows: injector(s) at 250 °C, carrier gas (hydrogen) at 25 psi (constant pressure), detectors at 320 °C and

make-up gas flow (nitrogen) at 25 mL/min. The oven temperature program was as follows: injection at 110 °C (hold for one minute), ramp temperature by 25 °C/min to 210 °C (hold for five minutes), ramp temperature by 5 °C/min to 240 °C (no hold) and, finally, ramp by 15 °C/min to 330 °C (hold for six minutes). The analytical signals were recorded by ChemStation Open Lab CDS.

### *1.2. Assessment of PCBs and OC Pesticides in Cord Blood Serum*

Positive identification of PCB congeners and OC pesticides was accomplished by retention time verification and peak shape analysis. The secondary column from the dual-column analysis was used for the confirmation of positively-identified peaks. Peaks that did not meet the retention time and peak shape criteria or were not confirmed by the secondary column were reported as non-numerical, interference peaks and did not contribute to the positive PCB and OC pesticide results and totals.

Quantification of PCB congeners and OC pesticides in the serum samples was achieved using multi-point calibration curves. The limits of detection (LODs) were established for each analyte using Equation (S1). The wet weight PCB congener and OC pesticide concentrations were also adjusted for lipid weight and sample volume using Equation (S2) [9–13].

$$\mathrm{LOD_{analyte}} = \frac{\text{analyte concentration in lowest calibrator}}{\text{sample volume}} \quad \text{(S1)}$$

$$\text{Lipid-adjusted analyte} = \frac{\text{analyte concentration} \times \text{sample volume}}{\text{sample lipid weight}} \quad \text{(S2)}$$

### *1.3. Laboratory Quality Control*

The MDHHS laboratory adhered to strict internal and external quality assurance/quality control (QA/QC) practices throughout the entire analytical procedure. The laboratory regularly participates in proficiency testing through the Arctic Monitoring and Assessment Programme (AMAP) and evaluates performance competencies in accordance with College of American Pathologist (CAP) and Clinical Laboratory Improvement Amendments (CLIA) regulations. In addition, the MDHHS laboratory maintains a fulltime QA manager who is responsible for ensuring that QA/QC practices are followed.

The following four levels of control materials were processed with each analytical batch of samples to measure extraction and cleanup efficiency and to monitor analytical accuracy and precision: QC blank (fetal bovine serum), QC low (National Institute of Standards and Technology (NIST) Standard Reference Materials (SRM) 1957), QC medium (NIST SRM 1958) and QC high (fortified fetal bovine serum). A surrogate spiking solution was added to all QCs and samples prior to extraction to verify the overall extraction efficiency and method of recovery. Instrument performance was monitored throughout the analytical run with calibration verification standards; an initial calibration verification standard (ICV) was analyzed immediately after the curve to verify linearity, while the continuing calibration verification (CCV) standard was dispersed throughout and at the end of the analytical run.

## References


1. U.S. Environmental Protection Agency. *Summary of the Toxic Substances Control Act*; U.S. Environmental Protection Agency: Washington, DC, USA, 1976.
2. Folch, J.; Lees, M.; Sloane Stanley, G.H. A simple method for the isolation and purification of total lipides from animal tissues. *J. Biol. Chem.* **1957**, *226*, 497–509.
3. Burse, V.W.; Needham, L.L.; Korver, M.P.; Lapeza, C.R., Jr.; Liddle, J.A.; Bayse, D.D. Assessment of methods to determine PCB levels in blood serum: Interlaboratory study. *J. Assoc. Off. Anal. Chem.* **1983**, *66*, 40–45.
4. Burse, V.W.; Head, S.L.; Korver, M.P.; McClure, P.C.; Donahue, J.F.; Needham, L.L. Determination of selected organochlorine pesticides and polychlorinated biphenyls in human serum. *J. Anal. Toxicol.* **1990**, *14*, 137–142.

5. Needham, L.L.; Burse, V.W.; Price, H.A. Temperature-programmed gas chromatographic determination of polychlorinated and polybrominated biphenyls in serum. *J. Assoc. Off. Anal. Chem.* **1981**, *64*, 1131–1137.
6. Needham, L.L. *Polychlorinated Biphenyl Determination at Parts-Per-Billion Level in Serum, CDC Laboratory Update*; US Department of Health and Human Services, Public Health Service, Centers for Disease Control: Atlanta, GA, USA, 1987.
7. Agilent Technologies. OpenLAB CDS ChemStation Edition. Available online: http://www.agilent.com/home (accessed on 16 June 2016).
8. Scion Instruments. Galaxie Chromatography Software (CompassCDS). Available online: https://scioninstruments.com/solutions/compasscds/ (accessed on 16 June 2016).
9. Phillips, D.L.; Pirkle, J.L.; Burse, V.W.; Bernert, J.T., Jr.; Henderson, L.O.; Needham, L.L. Chlorinated hydrocarbon levels in human serum: Effects of fasting and feeding. *Arch. Environ. Contam. Toxicol.* **1989**, *18*, 495–500.
10. Schisterman, E.F.; Whitcomb, B.W.; Louis, G.M.; Louis, T.A. Lipid adjustment in the analysis of environmental contaminants and human health risks. *Environ. Health Perspect.* **2005**, *113*, 853–857.
11. Bernert, J.T.; Turner, W.E.; Patterson, D.G., Jr.; Needham, L.L. Calculation of serum ”total lipid” concentrations for the adjustment of persistent organohalogen toxicant measurements in human samples. *Chemosphere* **2007**, *68*, 824–831.
12. Gaskins, A.J.; Schisterman, E.F. The effect of lipid adjustment on the analysis of environmental contaminants and the outcome of human health risks. *Methods Mol. Biol.* **2009**, *580*, 371–381.
13. Aminov, Z.; Haase, R.F.; Pavuk, M.; Carpenter, D.O. Analysis of the effects of exposure to polychlorinated biphenyls and chlorinated pesticides on serum lipid levels in residents of Anniston, Alabama. *Environ Health* **2013**, *12*, 108.

© 2016 by the authors. Submitted for possible open access publication under the terms and conditions of the Creative Commons Attribution (CC-BY) license (http://creativecommons.org/licenses/by/4.0/).
